# Detection of *HER2* amplification in circulating free DNA in patients with breast cancer

**DOI:** 10.1038/bjc.2011.89

**Published:** 2011-03-22

**Authors:** K Page, N Hava, B Ward, J Brown, D S Guttery, C Ruangpratheep, K Blighe, A Sharma, R A Walker, R C Coombes, J A Shaw

**Affiliations:** 1 Department of Cancer Studies and Molecular Medicine, University of Leicester, Robert Kilpatrick Building, PO Box 65, Leicester Royal Infirmary, Leicester LE2 7LX, UK; 2 Department of Oncology, Imperial College, MRC Cyclotron Building, Hammersmith Hospitals Trust, Hammersmith Hospital, Du Cane Road, London W12 ONN, UK; 3 Department of Medical Oncology, Charing Cross Hospital, London W6 8RF, UK

**Keywords:** breast cancer, tumour markers, *HER2* amplification, quantitative PCR

## Abstract

**Background:**

*Human epidermal growth factor receptor 2* (*HER2*) is amplified and overexpressed in 20–25% of breast cancers. This study investigated circulating free DNA (cfDNA) for detection of *HER2* gene amplification in patients with breast cancer.

**Methods:**

Circulating free DNA was extracted from plasma of unselected patients with primary breast cancer (22 before surgery and 68 following treatment), 30 metastatic patients and 98 female controls using the QIAamp Blood DNA Mini Kit (Qiagen). The ratio of *HER2* to an unamplified reference gene (*contactin-associated protein 1* (*CNTNAP1*)) was measured in cfDNA samples by quantitative PCR (qPCR) using SK-BR-3 cell line DNA as a positive control.

**Results:**

We validated the qPCR assay with DNA extracted from 23 HER2 3+ and 40 HER2-negative tumour tissue samples; the results agreed for 60 of 63 (95.2%) tumours. Amplification was detected in cfDNA for 8 of 68 patients following primary breast cancer treatment and 5 of 30 metastatic patients, but was undetected in 22 patients with primary breast cancer and 98 healthy female controls. Of the patients with amplification in cfDNA, 10 had HER2 3+ tumour status by immunohistochemistry.

**Conclusions:**

The results demonstrate for the first time the existence of amplified *HER2* in cfDNA in the follow-up of breast cancer patients who are otherwise disease free. This approach could potentially provide a marker in patients with HER2-positive breast cancer.

The *human epidermal growth factor receptor 2* gene encodes a 185 kd cell surface glycoprotein (*HER2*), with intrinsic tyrosine kinase activity, that plays a crucial role in regulating cell growth and differentiation (Ullrich and Schlessinger, 1990). The protein is overexpressed because of gene amplification in 20–25% of breast cancers (Slamon *et al*, 1987), where it is a diagnostic marker for aggressive disease and increased risk of metastasis and thus poor prognosis (Ross and Fletcher, 1998). Over expression of HER2 is not found in normal breast tissue or in benign breast lesions, but is found in ∼30% of ductal carcinomas *in situ* (DCIS) (van de Vijver *et al*, 1988). Breast cancer patients can be successfully treated with the humanised monoclonal antibody trastuzumab (Herceptin, Hoffman-La Roche Ltd, Basel, Switzerland) (Pegram *et al*, 1998), and therefore accurate evaluation of HER2 status is essential for better management.

Currently, two methods of determining HER2 status are used routinely in clinical practice: immunohistochemistry (IHC), which assesses expression of the HER2 protein on the cell surface, and fluorescent *in situ* hybridisation (FISH), which detects amplification of the *HER2* gene (Pauletti *et al*, 2000; Bartlett *et al*, 2001; Lebeau *et al*, 2001). Results can be affected by tissue fixation, particularly for IHC and require standardised protocols with quality assurance. Both of these methods require tumour biopsies, removed by invasive techniques, and do not allow the monitoring of minimal residual disease on follow-up. Patients are more likely to respond to trastuzumab treatment for metastatic disease if they have *HER2* amplification than if they have HER2 overexpression without amplification. Therefore, methods that detect copy number increase may be better than protein overexpression assays such as IHC (Vogel *et al*, 2001).

It was initially thought that *HER2* amplification and overexpression were stable over the course of disease and concordant between primary tumour and metastases, and therefore patients with low levels of expression at first presentation of breast cancer are rarely given anti-HER2 (trastuzumab) treatment. However, in 2004, Meng *et al* (2004) demonstrated that *HER2* gene amplification can be acquired as breast cancer progresses; this concurs with results using immunocytochemistry of circulating tumour cells (Wulfing *et al*, 2006) showing that 50% of patients had evidence of HER2-positive cells in the blood and that this correlated with survival; the proportion of positive cells exceeded that expected by IHC of the primary tumour. Quantitative PCR (qPCR) has been used to determine *HER2* amplification status in tumour DNA and has shown similar results to FISH, and some discordance with tissue-based IHC (Kulka *et al*, 2006; Monego *et al*, 2007); however, the method has not been applied previously to circulating free DNA (cfDNA).

It is well established that higher concentrations of cfDNA can be found in the blood of patients with cancer than in healthy people (Goebel *et al*, 2005). However, this is not cancer specific as elevated levels of cfDNA are also detected in patients with other pathological conditions such as systemic lupus erythematosus, rheumatoid arthritis and other conditions associated with inflammatory processes (Davis and Davis, 1973; Koffler *et al*, 1973; Leon *et al*, 1981). A variety of methods are used for isolation and quantitation of circulating DNA, with the Qiagen QIAamp Blood Mini Kit (Qiagen, Hilden, Germany) and real-time PCR quantification being most common (van der Vaart and Pretorius, 2010). Although most studies of cfDNA suggest that apoptosis and/or necrosis are the predominant sources of cfDNA, active release of cfDNA by living cells, and disturbances in the clearance of this, are also plausible mechanisms (van der Vaart and Pretorius, 2008). In this study, we developed a qPCR assay to detect *HER2* amplification in cfDNA. We compared *contactin-associated protein 1* (*CNTNAP1*), *glyceraldehyde-3-phosphate dehydrogenase* (*GAPDH*) and *ribonuclease P RNA component H1* (*RPPH1*) as unamplified reference genes and showed that all three were appropriate for measuring *HER2* amplification using breast cancer cell lines and normal DNA controls. We obtained blood from unselected primary breast cancer patients, patients on follow-up following primary breast cancer treatment, metastatic cases and women with benign breast disease and healthy female controls and analysed cfDNA for the presence of amplified *HER2* DNA. We also compared plasma results in 10 patients at the start of herceptin treatment and while on therapy. The results suggest that plasma may be used as a surrogate for a tumour biopsy in some patients.

## Materials and methods

### Patients and samples

The protocols were approved by the Riverside regional ethics committee and conducted in accordance with the Declaration of Helsinki. All patients gave written informed consent before participation. Samples were blinded for analysis and patients understood that the results would not be made available to them. After obtaining statistical advice, we collected and analysed blood samples from 22 women attending clinic who had just been diagnosed with primary breast cancer, 6 patients with DCIS, 39 patients with benign breast disease and from 59 healthy female volunteers. We also retrospectively analysed stored plasma samples from 78 primary breast cancer patients on follow-up following surgery, from a previously published cohort (Slade *et al*, 2009) and 30 patients with overt metastases (Table 1).

Following plasma separation by centrifugation at 850 **g** for 10 min ( × 2), plasma and cell pellets were separated and stored directly at −80 °C. On thawing, plasma was centrifuged at 1500 **g** for 5 min and the supernatant was removed to a clean tube before isolation of DNA.

### DNA extraction

DNA samples used for assay development and validation were from breast cancer cell lines MDA-MB-231 and SK-BR-3 (obtained from ATCC, Manassas, VA, USA) and human genomic DNA (Roche Applied Science, West Sussex, UK), comprising a pool of ∼100 blood donors from both sexes.

Lymphocyte and plasma cfDNA were extracted using the QIAamp Blood DNA Mini Kit (Qiagen) according to the blood and body fluid protocol as described previously (Page *et al*, 2006; Shaw *et al*, 2011).

For analysis of tumour samples, 5 *μ*m haematoxylin and eosin-stained formalin-fixed and paraffin-embedded (FFPE) tissue sections were reviewed by a pathologist and foci of tumour cells were isolated by manual microdissection. DNA was extracted by standard proteinase K digestion, phenol–chloroform extraction and ethanol precipitation. DNA pellets were re-suspended in 50 *μ*l sterile ultrapure water.

### Quantitative PCR detection of *HER2* gene amplification

Primers and a FAM-labelled minor groove binder (MGB) TaqMan probe were targeted to *HER2* (target; locus 17q21.1), *CNTNAP1* (unamplified reference (based on data available at the start of the study at http://www.sanger.ac.uk/genetics/CGP/cosmic), locus 17q21) and *GAPDH* (unamplified reference*,* locus 12p13.1), as described previously (Shaw *et al*, 2011), and *RPPH1* (unamplified reference, locus 14q21) (forward primer: 5′-CGGAGGGAAGCTCATCAGTG-3′, reverse primer: 5′-GACATGGGAGTGGAGTGACA-3′, MGB probe: 5′-CACGAGCTGAGTGCGT-3′). All assays were carried out in triplicate on MicroAmp Fast plates (Applied Biosystems, Foster City, CA, USA) in a 10 *μ*l reaction volume comprising: 5 *μ*l TaqMan Universal Fast Mastermix (Applied Biosystems), 600 nmol forward and reverse primers, 200 nmol FAM-MGB TaqMan probe and 3.6 *μ*l template DNA. A human genomic DNA sample (Roche Applied Science) was included in each experiment together with SK-BR-3 cell line DNA (as a positive control for *HER2* amplification) and sterile water as a no template control. Reactions were run on Applied Biosystems thermal cyclers (Step One and 7900 Fast) with an initial activation step at 95 °C for 20 s followed by 50 cycles of 95 °C for 1/3 s and 60 °C for 20/30 s (Step One/7900 Fast).

To determine *HER2* gene amplification, the ΔCt was determined (average Ct value of the target gene minus the average Ct value of the reference gene) and used to calculate the ΔΔCt for each DNA sample, using the mean relative quantitation (RQ) value derived from a panel of 49 normal lymphocyte DNA samples (RQ=1.003±0.086) as the experimental calibrator. The RQ values were calculated as 2^−ΔΔCt^. The *Z*-score was calculated as follows: *Z*-score=(ΔCt patient sample−ΔCt average normal lymphocyte panel)/average s.d. of normal lymphocyte panel. A negative *Z*-score value indicated significant amplification (−3.30=99.9% confidence, −2.60=99% confidence and −1.96=95% confidence).

Statistical analysis was performed using GraphPad Prism version 5.0 (San Diego, CA, USA). Unpaired *t*-tests, followed by Mann–Whitney tests, were used to compare two patient groups. The Dunn's multiple comparison test was used to compare s.d. values from all cfDNA samples.

### HER2 immunohistochemistry

HER2 status was obtained from the patient records where known. The HER2 status of the primary tumour was not known for 63 of the 130 patients as their diagnosis predated the introduction of HER2 testing in the United Kingdom in October 2006. Immunohistochemistry was performed using the monoclonal antibody CB11 (Novocastra, Newcastle, UK) following established guidelines (Walker *et al*, 2008). All IHC slides were reviewed by one of the authors (RAW).

## Results

### Reference gene selection and validation of *HER2* amplification in cell lines and FFPE tumour DNA

**Figure 1:**
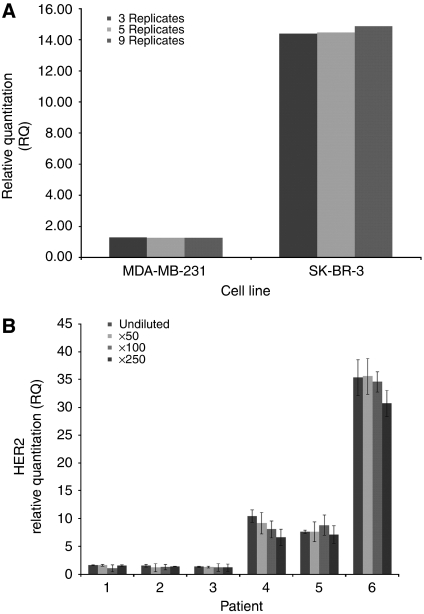
Validation of *HER2* amplification in cell lines and FFPE tumour DNA. (**A**) Mean *HER2* RQ for SK-BR-3 (*HER2* amplified) and MDA-MB-231 (unamplified) cell line DNA, measured using 3, 5 and 9 independent replicates. (**B**) Mean *HER2* RQ for three HER2 IHC-negative (patients 1–3) and three HER2 3+ (patients 3–6) tumour DNA samples. DNA was diluted 50-, 100- and 250-fold. Bars show the mean RQ at each dilution of DNA±s.d.

We first measured the ratio of *HER2* to three separate reference loci, *CNTNAP1*, *RPPH1* and *GAPDH*, in a panel of 49 lymphocyte DNA samples isolated from healthy female controls. The mean RQ values were 1.102±0.358 for *CNTNAP1*, 1.000±0.120 for *GAPDH* and 0.565±0.300 for *RPPH1*, confirming a 1 : 1 ratio of *HER2* to *CNTNAP1* and *GAPDH* in normal lymphocyte DNA samples. We next surveyed DNA isolated from two breast cancer cell lines of known *HER2* amplification status (SK-BR-3 (amplified) and MDA-MB-231 (unamplified)), using the qPCR assay. SK-BR-3 showed high amplification (mean RQ=14.3) and MDA-MB-231 showed no amplification (mean RQ=0.8). These results were reproducible using 10 ng of genomic DNA, when the starting DNA was diluted 250-fold (data not shown), and over 3, 5 and 9 independent replicates (Figure 1A).

We next validated the assay using DNA isolated by microdissection from FFPE tissue sections from 63 tumours: 23 were scored as HER2 3+ and 40 as HER2 negative by IHC. RQ values of ⩾2.0 (Suo *et al*, 2004) and ⩾2.7 (Kulka *et al*, 2006) have been considered previously to be positive for *HER2* gene amplification. As RQ values of ⩾2.1 all showed negative *Z*-scores of ⩽−3.30 (99.9% confidence interval), we selected this as the cutoff for *HER2* amplification. The RQ results agreed with the IHC results for 60 (95.2%) of the 63 samples. Discordant findings were for one HER2 3+ tumour that showed no amplification in the corresponding DNA (RQ=1.84) and two tumours, reported as HER2 negative by IHC, that showed amplification in microdissected foci of tumour cells (RQ values of 2.31 and 2.22, respectively). Results were consistent when the tumour DNA was diluted 250-fold (Figure 1B).

### Detection of *HER2* gene amplification in cfDNA

**Figure 2:**
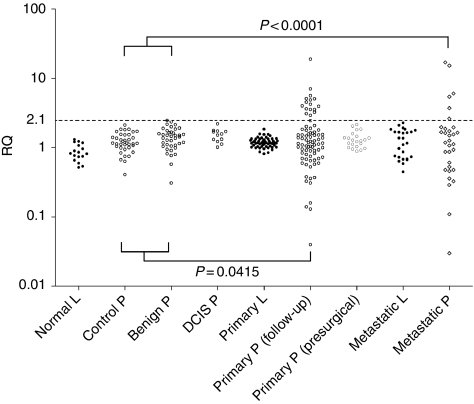
Detection of *HER2* gene amplification in cfDNA of breast cancer patients. Lymphocyte (L) and plasma (P) cfDNA RQ values for all samples using 49 normal lymphocyte DNA samples as a reference panel to calculate ΔΔCt and hence RQ. Unpaired *t*-tests were performed to compare pooled controls and benign samples with both primary and metastatic patient groups.

We first screened cfDNA from 59 healthy female controls, 39 patients with benign breast disease, 6 cases of DCIS and 63 normal lymphocyte DNA samples. None showed RQ values of ⩾2.1, our cutoff for *HER2* amplification, although the RQ value was ∼2.0 for cfDNA from one healthy control and two benign breast disease patients, indicating potential amplification. None of the 22 untreated primary breast cancer patients had *HER2* amplification in cfDNA, although only two had a HER2-positive tumour by IHC. Clear evidence of *HER2* amplification was detected in cfDNA of 13 breast cancer patients: 8 of 78 patients on follow-up following primary breast cancer treatment and 5 of 30 metastatic patients (Figure 2). The mean RQ value for the 13 positive cfDNA samples was 5.56, well above the RQ threshold. The RQ values were very similar using results from both a panel of 49 normal lymphocyte DNA samples and commercially available Human Genomic DNA to calculate ΔΔCt and hence RQ. Although RQ values of cfDNA samples derived from breast cancer patients were spread over a broader range than for other samples (Figure 2), Dunn's multiple comparison test showed no significant difference between the s.d. values of patients and controls. When we compared the number of samples with amplification in each group, there was a statistically significant difference between pooled healthy and benign patient samples and both primary patients on follow-up and metastatic patients (*P*=0.0415 and <0.0001, respectively, Figure 2).

### Comparison of cfDNA with tumour phenotype

**Figure 3:**
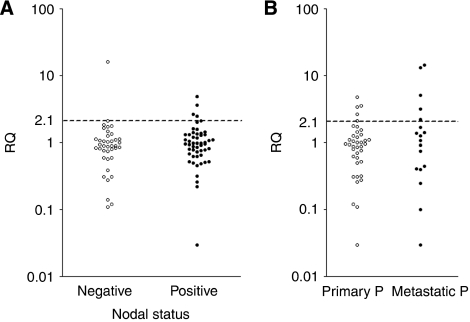
Relationship between *HER2* amplification in cfDNA and patient node and HER2 3+ tumour status. (**A**) Circulating free DNA RQ values for primary patients on follow-up by node status. (**B**) Circulating free DNA RQ values for all primary patients on follow-up and metastatic patients with HER2 3+ tumour status by IHC.

We reviewed all patient records for the HER2 status of the primary tumour and performed IHC in 63 cases for whom FFPE tissue was available. In total, there were 39 of 90 primary patients (22 presurgical and 68 on follow-up) and 18 of 30 metastatic patients with a HER2 3+ tumour status by IHC. When we compared cfDNA results with the corresponding primary tumour IHC, we found that six of the eight primary patients on follow-up and all five metastatic patients with amplification in cfDNA had HER2 3+ tumour status (Figure 3A). However, not all patients with HER2 3+ tumour status showed amplification in cfDNA. There were 2 presurgical primary breast cancer patients, 31 primary breast cancer patients on follow-up and 13 patients with overt metastases whose tumours were HER2 3+ who had negative cfDNA tests. There were also two primary patients on follow-up and one metastatic patient with HER2 2+ tumour status who had a negative cfDNA test. In the eight primary patients on follow-up, we next compared patients with amplification in cfDNA with node status: 6 were node positive and 2 were node negative (Figure 3B). There was no association between patients with *HER2* amplification in cfDNA and any other clinicopathological variable. There were 12 primary breast cancer patients and 12 patients with metastatic disease who had completed trastuzumab therapy before venepuncture, and all were negative for *HER2* amplification in cfDNA apart from 3 metastatic breast cancer patients who had RQ values of 3.71, 6.02 and 16.97 in cfDNA (Table 2). To investigate this further, we recruited 10 patients, before commencing treatment with trastuzumab, and compared paired blood samples for these taken before treatment and 3 to 4 months after starting therapy. Of the 10 patients, 6 had a positive plasma test by RQ, which was consistent both before treatment and while on therapy, and 4 patients were negative by RQ in both samples.

## Discussion

This study investigated the potential utility of cfDNA as a source for detection of *HER2* gene amplification in patients with breast cancer. The mean RQ value achieved by normal diploid lymphocyte samples was 1.08 by the assay we have used and therefore we chose to use RQ ⩾2.1 for *HER2* amplification in order to reduce the chances of any false-positive results (Suo *et al*, 2004). The RQ assay was specifically able to detect *HER2* gene amplification, as the reference gene chosen (*CNTNAP1*) lies on the same chromosome as *HER2*. There is clearly potential for changes in copy number in tumour DNA, and hence it would be preferable to use a panel of reference genes to detect amplification, but limiting cfDNA quantities prevent using such an approach. Our RQ assay showed good correlation with results from IHC (95.2%), using DNA isolated by microdissection from 63 FFPE tumour tissue samples. Moreover, the assay was reproducible between replicate experiments, and when using a 250-fold dilution of tumour DNA. Where amplification was detected, it was specific for *HER2* rather than polysomy 17, as RQ values were similar using both *CNTNAP1* and *GAPDH* as the reference genes (data not shown). Polysomy 17 has been shown to have no effect on *HER2* mRNA levels or protein expression (Dal Lago *et al*, 2006; Fehm *et al*, 2007).

The results have shown that it is possible to detect *HER2* amplification in cfDNA of some patients on follow-up following primary breast cancer treatment despite the fact that they had no evidence of metastatic disease, either at the time of sampling or >6 years after. This is in sharp contrast to the 39 patients with benign breast disease and 59 healthy women, who showed no evidence of *HER2* amplification in cfDNA using a cutoff for RQ of 2.1. Of the 78 breast cancer patients on follow-up after primary treatment, 12 had received trastuzumab before venepuncture. In these patients, previous trastuzumab therapy was associated with a negative plasma qPCR test. Of the 30 patients with metastatic disease, 12 had received trastuzumab treatment: 10 of these were negative by RQ and 2 had RQ values of 6.02 and 16.97 in cfDNA (Table 2). We also compared cfDNA results in 10 primary breast cancer patients before commencement of trastuzumab treatment and while on therapy. All 10 patients showed consistent results between their paired samples (6 were positive by RQ and 4 were negative). Together, the results suggest that a change from positive to negative RQ results might indicate successful therapy, as was suggested in a recent study of 28 patients (Sorensen *et al*, 2010). However, the findings need confirmation in a larger series, comparing RQ results for cfDNA pre- and post-trastuzumab therapy and with clinical response.

Not all patients with HER2 3+ tumours by IHC had detectable gene amplification in cfDNA. This is perhaps not surprising as a majority of patients were sampled several years after removal of their primary breast cancer and therefore may have been cured. For the eight primary breast cancer patients with *HER2* amplification in cfDNA, the follow-up time is insufficient to inform us as to whether the presence of circulating amplified *HER2* DNA is predictive of recurrent disease. Other studies have shown that some patients, whose tumours were HER2 negative, show circulating tumour cells (CTCs) that express HER2, and circulating HER2 extracellular domain (ECD) is also found in similar groups of patients (Fehm *et al*, 2007). In one study, many patients with metastatic disease had CTCs expressing HER2, but most of these patients were end stage with extensive metastatic disease, and had received multiple courses of chemotherapy (Vogel *et al*, 2001). The authors suggest that chemotherapy exerts a selective pressure, allowing the emergence of HER2-positive cells. Several groups have now presented evidence that HER2-positive CTCs can be detected in patients with HER2-negative primary breast cancer during follow-up (Braun *et al*, 2001; Zidan *et al*, 2005; Wulfing *et al*, 2006; Fehm *et al*, 2010). The data presented in this publication support these findings, as *HER2-*amplified cfDNA was detected in two HER2-negative patients following primary breast cancer treatment. It has been reported that this indicates a poor prognosis (Wulfing *et al*, 2006); it is not clear whether these cells represent a switch in phenotype or a subgroup of pre-existing cells within the primary tumour.

**Figure 4:**
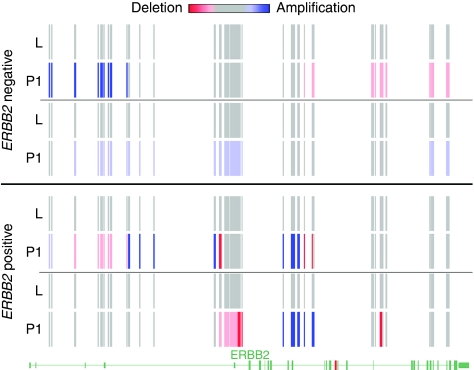
Copy number variation in cfDNA in the *HER2* gene interval detected by SNP 6.0 Array. Top panel paired plasma and leukocyte DNA samples for 2 HER2 IHC-negative patients, and lower panel paired plasma and leukocyte DNA samples for 2 HER2 3+ patients. Each vertical bar represents one copy number (CN) marker and its value is as inferred by the colour scale, with blue showing amplification and red showing deletion. The exons and introns of the *HER2* gene are indicated at the bottom of the diagram, with the location of the qPCR assay (exon 16) marked in red. L, leukocyte DNA; P1, plasma cfDNA.

We have recently compared cfDNA and CTC detection in 19 other patients on follow-up (median 3.5 years) (Shaw *et al*, 2011). Of these, 17 demonstrated no evidence of CTCs, 1 patient had 1 CTC and 1 patient had 3 CTCs. Also, eight patients had HER2 3+ tumours and four of these had HER2 amplification in cfDNA, but these did not overlap with the two CTC-positive patients. None of the patients have relapsed thus far, but results in this separate cohort confirm that HER2 amplification is detectable in plasma of patients on follow-up. We have also analysed cfDNA of 65 breast cancer patients for copy number variations using Affymetrix (High Wycombe, UK) SNP 6.0 Array (JA Shaw *et al*, in preparation). In all, 50 patients were on follow-up and overlap with patients in this study. The normal leukocyte DNA samples and cfDNA of 37 HER2-negative patients showed mostly diploid copy number (mean copy number (CN) state=2.0), whereas plasma samples of 13 HER2 3+ patients showed a mean CN state of 2.5–3.0 by smooth signal, indicating a low level of amplification (Figure 4). Hence, we have confirmed the ability to detect *HER2* amplification in cfDNA by another method.

In conclusion, this study has demonstrated the existence of circulating amplified *HER2* DNA, both in the follow-up of breast cancer patients otherwise disease free and in patients with metastatic disease. The follow-up time is insufficient to inform us as to whether the presence of circulating amplified *HER2* DNA is predictive of recurrent disease. The results in cfDNA do not always correlate with the primary tumour, suggesting that *HER2* gene amplification can be acquired as breast cancer progresses due to emergence of HER2-positive cells. Future studies will focus on examining the relationship between cfDNA and CTC *HER2* expression and determining the reason for changes in *HER2* status over time; we also hope to establish the prognostic significance of finding circulating *HER2-*amplified DNA in patients on follow-up and compare the different methods for monitoring minimal residual disease during adjuvant therapy in patients with breast cancer.

## Figures and Tables

**Table 1:** Characteristics of the primary tumour from breast cancer patients

	**Primary breast cancer following treatment**		**Pre-treatment primary breast cancer**		**Metastatic breast cancer**	
	**No.**	**%**	**No.**	**%**	**No.**	**%**
*Histology*						
Invasive ductal	55	71	13	59	26	87
Invasive lobular	8	10	4	18	3	10
Other/mixed invasive	15	19	5	23	1	3
						
*Lymph node status*						
Positive	48	62	6	27	19	63
Negative	29	37	12	55	5	17
Unknown	1	1	4	18	6	20
						
*Tumour size*						
T1	43	55	9	41	10	33.3
T2	24	31	8	36	7	23.4
T3	8	10	1	5	3	10
T4	0	0	0	0	0	0
Unknown	3	4	4	18	10	33.3
						
*Tumour grade*						
I	5	6	3	13.6	0	0
II	31	40	16	72.7	17	57
III	41	53	2	9.1	9	30
Unknown	1	1	1	4.5	4	13
						
*Hormone receptor status*						
ER positive	50	64	21	95	20	66.7
ER negative	28	36	1	5	10	33.3
PgR positive	34	43	21	95	16	54
PgR negative	39	51	1	5	12	40
Unknown PgR status	5	6	0	0	2	6
						
*HER2 status*						
Positive	37	47	2	9	18	60
Negative	41	53	20	91	12	40
Total	78	100	22	100	30	100

Abbreviations: ER=estrogen; PgR=progesterone; HER2=human epidermal growth factor receptor 2. The American Joint Committee on Cancer (AJCC) staging system was used to classify tumour size.

**Table 2:** *HER2* RQ results in tumour and cfDNA of 30 metastatic breast cancer patients

		**Trastuzumab before blood sample**		**RQ value**	
**Patient**	**HER2 IHC**	**(disease status Jan 2011)**	**Date of blood sample**	**Plasma**	**Tumour**
1	3+	No	08/2008	0.87	NA
2	3+	Yes	05/2007	6.02	35.30
3	3+	Yes (S)	04/2007	0.29	3.72
4	3+	Yes	2002	0.48	23.96
5	3+	No	07/2008	1.66	4.23
6	3+	Yes (S)	04/2007	0.53	NA
7	3+	Yes (L)	03/2007	0.11	16.58
8	3+	Yes	03/2007	16.97	6.52
9	3+	Yes	03/2007	3.71	81.74
10	3+	Yes	05/2007	0.03	NA
11	3+	Yes	2002	1.62	2.24
12	3+	No	2002	2.61	7.99
13	3+	Yes	2002	0.46	9.15
14	3+	No	2002	15.28	4.76
15	3+	Yes	07/2008	1.26	22.60
16	3+	Yes	07/2008	1.49	1.99
17	Neg	Unknown	07/2008	1.18	NA
18	Neg	Unknown (P)	08/2008	1.95	NA
19	Neg	Unknown	08/2008	5.41	2.10
20	2+	Unknown	08/2008	1.86	2.42
21	Neg	Unknown	2001	0.33	0.85
22	Neg	No	2002	0.87	0.83
23	Neg	No	2002	0.61	8.53
24	Neg	No	2002	0.93	1.10
25	Neg	No	2002	0.36	NA
26	Neg	No	2002	1.73	NA
27	Neg	No (P)	04/2007	1.54	NA
28	Neg	No	06/2007	1.12	NA
29	3+	No (H)	08/2009	1.07	NA
30	3+	No (H)	08/ 2009	1.96	NA

Abbreviations: HER2=human epidermal growth factor receptor 2; RQ=relative quantitation; cfDNA=circulating free DNA; IHC=immunohistochemistry; Neg=negative; NA=no tissue available. Disease status: S=stable; L=lapatinib; P=disease progressing; H=adjuvant herceptin. Where no disease status indicated, deceased.

Showing RQ value for DNA extracted from microdissected foci of the primary tumour and from cfDNA.
